# The Determinants of B Cell Receptor Signaling as Prototype Molecular Biomarkers of Leukemia

**DOI:** 10.3389/fonc.2021.771669

**Published:** 2021-12-21

**Authors:** Antonella Nicolò, Alexandra Theresa Linder, Hassan Jumaa, Palash Chandra Maity

**Affiliations:** Institute of Immunology, Ulm University, Ulm, Germany

**Keywords:** BCR signaling, CLL, biomarkers, transformation, immunoglobulin genes

## Abstract

Advanced genome-wide association studies (GWAS) identified several transforming mutations in susceptible loci which are recognized as valuable prognostic markers in chronic lymphocytic leukemia (CLL) and B cell lymphoma (BCL). Alongside, robust genetic manipulations facilitated the generation of preclinical mouse models to validate mutations associated with poor prognosis and refractory B cell malignancies. Taken together, these studies identified new prognostic markers that could achieve characteristics of precision biomarkers for molecular diagnosis. On the contrary, the idea of augmented B cell antigen receptor (BCR) signaling as a transforming cue has somewhat receded despite the efficacy of Btk and Syk inhibitors. Recent studies from several research groups pointed out that acquired mutations in BCR components serve as faithful biomarkers, which become important for precision diagnostics and therapy, due to their relevant role in augmented BCR signaling and CLL pathogenesis. For example, we showed that expression of a single point mutated immunoglobulin light chain (LC) recombined through the variable gene segment IGLV3-21, named IGLV3-21^R110^, marks severe CLL cases. In this perspective, we summarize the molecular mechanisms fine-tuning B cell transformation, focusing on immunoglobulin point mutations and recurrent mutations in tumor suppressors. We present a stochastic model for gain-of-autonomous BCR signaling and subsequent neoplastic transformation. Of note, additional mutational analyses on immunoglobulin heavy chain (HC) derived from non-subset #2 CLL IGLV3-21^R110^ cases endorses our perspective. Altogether, we propose a model of malignant transformation in which the augmented BCR signaling creates a conducive platform for the appearance of transforming mutations.

## Introduction

**Figure 1:**
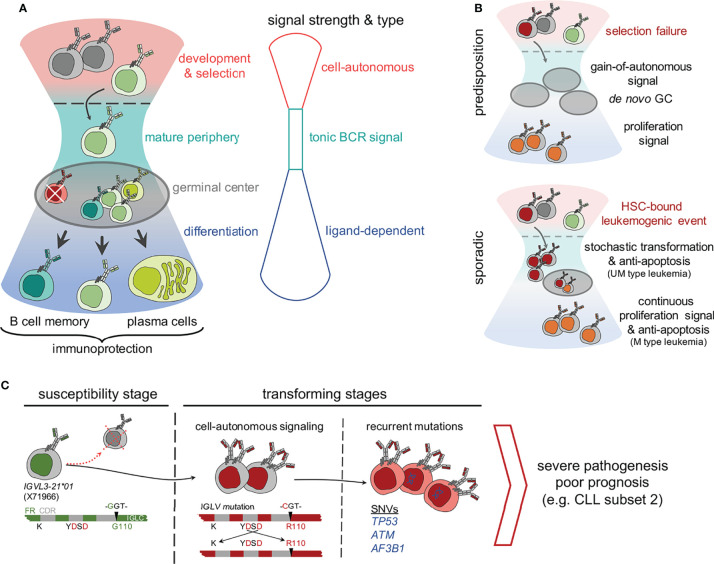
Compounding effect of molecular determinants of BCR signaling and recurrent mutations in genetically predisposed and sporadic origin CLL. **(A)** A schematic hourglass portrays the course of B cell development, selection, peripheral maintenance, activation and differentiation regulated through tight controls and varying strength of BCR signaling. As shown, each stage represents a predominant type of BCR (or pre-BCR) signaling, which controls the outcome as follows, 1) cell-autonomous signaling at bone marrow dwelled development and selection, 2) tonic signal at the mature peripheral compartments, and 3) antigen responsiveness or ligand-dependent activation signal for memory and plasma cell differentiation during the germinal center reaction. **(B)** Mutations that supersede the balance between BCR signal strength and developmental stages are a threat for genetic predisposition of leukemia. For example, a selection failure or gain-of-autonomous signaling mutation result in persisting autonomously active B clones in the peripheral compartment that promote proliferative boosts and prime *de novo* germinal center (GC) formations. In contrast, sporadic mutations have diverse origins. For example, a leukemogenic event occurring at haemopoietic stem cells (HSC) undergoing stochastic transformation might lead to anti-apoptosis and proliferation boost. **(C)** A stochastic model of neoplastic transformation through acquisition of biomarkers on susceptible genetic background exemplified by the acquired IGLV3-21^R110^ mutated CLL pathogenesis. As shown, individuals carrying the allele IGLV3-21*01 are predisposed to gain-of-autonomous BCR signaling through homotypic BCR : BCR interaction stabilized by IGLV3-21^R110^. Although mostly eliminated and undetected in the peripheral blood of a healthy IGLV3-21*01 carrier, a single point G→C mutation, possibly induced by activation-induced cytidine deaminase (AICDA) activity, converts a Glycine (G) to Arginine at 110th residue (R110) and initiates the neoplastic transformation. The gain-of-autonomous BCR signaling and enduring survival advocate the persistent single nucleotide variations (SNV’s) stochastically in *TP53*, *ATM*, splicing factor *SF3B1* and in epigenetic modifiers to culminate the leukemogenesis and develop severe CLL.

The B cell antigen receptor (BCR) signaling is the key survival and growth promoter for both normal and malignant B cells, controlling important cell fate decisions including proliferation and differentiation (1). Depending on quality, capacity and relevance, three different types of BCR signaling were described: cell-autonomous, tonic and standard ligand-dependent signaling (**Figure 1A**). B cells are engineered to control the BCR signaling strength and type during their development step-by-step (1–3). First, the autoreactive clones are self-eliminated by altered BCR activation threshold in the bone marrow compartment. Thereafter, tonic BCR signaling controls cell survival and proliferation, relying on the crosstalks integrating microenvironmental signals and cytoskeletal remodeling (1, 4). Finally, the antigen stimulated BCR signal promotes clonal expansion and differentiation during the germinal center (GC) reaction. A dysregulation of BCR signaling at any of the aforesaid levels, such as a gain-of-function mutation in signaling components, results in altered B cell survival, most often allowing escape from self-elimination process and resulting in primary immunodeficiencies, autoimmune diseases and B cell malignancies (**Figure 1B**) (5, 6). In particular, mutations or differential membrane organization of receptors which lead to the constitutive activation of the BCR are mainly associated with B cell malignancies, such as CLL and activated B cell-like diffuse large BCL (ABC DLBCL) (1, 2, 7, 8). Although sporadic and non-familial pathogenesis predominates in B cell neoplastic diseases, recent sequencing approaches and GWAS studies provided evidence of an inherited predisposition by identifying risk-associated loci in CLL and DLBCL (9–13). Recurrent mutations are mostly located in the non-coding regions of the genome, affecting active promoters or enhancers (12, 14). Very often, these recurrent genomic aberrations are functionally associated to genomic-instability, anti-apoptosis, and abnormal lymphoproliferation signaling networks (**Figure 1B**) (9, 11, 15, 16). Notably, the dysregulation of the Bcl-2 family of anti-apoptotic proteins, including Bcl-2, Bcl-xL and Mcl-1, permits escape from intrinsic and extrinsic apoptosis (17). Targeting this anti-apoptosis pathway using Bcl-2 inhibitors such as venetoclax, is a faithful alternative for treatment of resistant and refractory CLL as well as other B cell malignancies (18, 19). Along with identifying reliable signature mutations, continuous efforts have been made to analyze the leukemogenic potential of these sporadic mutations in genetically modified animals (20–23). For example, the *TP53* and *ATM* mutations worsen the CLL prognosis and B lineage specific deletions of these genes induce early disease onset and aggressive leukemic features in Eµ-TCL1 mice model (22, 23). Similarly, gain-of-function mutation in the splicing factor SF3B, when combined with *ATM* deletion, presents CLL-like B cells in mouse models (23). These results strongly imply the role of prognostically adverse mutations and chromosomal anomalies in CLL pathogenesis but are unable to define the role of BCR signaling in the transforming process.

Importantly, CLL prognosis is still strongly relying on the BCR and associated features, in particular on the mutational status, rearranged immunoglobulin genes and complementary determining region 3 (CDR3) (24, 25). These molecular determinants are potential functionally relevant biomarkers. Here, we review the current understanding of BCR associated biomarker exemplifying a novel IGL mutation, termed IGLV3-21^R110^ (where a glycine is replaced by arginine at position 110), in severe pathogenesis including stereotypic CLL subset #2 (26–28). Additionally, we show that the gain-of-autonomous BCR signaling requires point mutations in both IGLV and IGHV genes derived from a non-subset #2 CLL IGLV3-21^R110^ case. Thus, we postulate that the gain-of-autonomous BCR signaling and a convoluted signaling crosstalk might favor the recurrent mutations, thus contributing to genomic-instability and anti-apoptosis. Taken together, we endorse the use of BCR associated molecular biomarkers as a novel tool for an easy and comprehensive characterization of CLL.

## Identification of IGLV3-21^R110^ as a Model of Molecular Biomarker for Rapid Prognosis of Severe CLL

The clinical course of CLL varies widely, ranging from patients with stable, asymptomatic disease without need of therapeutic intervention to patients suffering from a progressive disease requiring immediate treatment after diagnosis (15, 24, 25, 29). CLL diagnosis is established by routine laboratory tests such as blood counts, blood smear, immunophenotyping, and assessment of the IGHV mutational status or chromatin aberration by FISH (15, 24). Notably, the IGHV mutational status is a first-line molecular determinant or biomarker for prognosis and classification of CLL with highly significant differences in clinical behavior (30, 31). Notably, a biomarker indicates the disease states or medical signs of patients. The first working definition of biomarker from the U.S. National Institutes of Health (NIH) and the World Health Organization (WHO) delineated the objective characteristics, i.e., accurate and reproducible indicators of pathogenic processes or responses to a therapeutic intervention (32, 33). Moreover, there are at least two subclasses of biomarkers, namely prognostic and predictive, based on interaction and differential response to treatment (34, 35). In the last twenty years, this broad objective definition was extended, diversified, and subclassified by several authors (36). Nevertheless, the improvements in precision medicine compel the search for new biomarkers. More recently, we focus on the mechanism-centric approaches for functionally meaningful biomarker selection (36). Unfortunately, identification of a functionally relevant biomarker, specifically for B cell malignancies, requires multifaceted validations, including the generation of mouse models, which makes it meticulous and challenging (20, 21, 37).

For many years, classical staging systems of Rai and Binet defined three major prognostic groups based on clinical parameters derived from physical examination and the aforementioned standard laboratory tests. However, with recent developments in CLL therapy and the sequencing techniques, a number of novel potential prognostic markers has been identified. Thus, the classical staging systems have become insufficient to distinguish prognostic groups and predict early disease progression (24, 38). To overcome these limits, a novel comprehensive prognostic score, the CLL international prognostic index (CLL-IPI) was created. The CLL-IPI combines biochemical and clinical parameters (age, clinical staging, serum ß-2 microglobulin) with cellular and genetic features such as *TP53* and IGHV mutational status, to provide a more advanced risk stratification (24, 39). Of the five prognostic factors used by CLL-IPI, *TP53* and IGHV mutation status have been described as particularly important in determining patient outcomes (15, 24, 39). In parallel, prognostic groups of stereotyped CLL subsets have also been defined according to the distinctive amino acid pattern within the IGHV CDR3 region (25, 40, 41). Nevertheless, not all CLL cases belong to stereotyped subsets, with roughly 60% of the CLL BCRs remaining unclassified (25, 41). Notably, neither the CLL-IPI grading system nor the subset classification considers the mutations in immunoglobulin light chain (LC), which are known to have direct impact on BCR signaling and prognostic relevance (26–28, 42). Intriguingly, most stereotypic BCR consist of an unique pair of HC and LC recombined through specific V(D)J segments (41, 43). Yet, the role of the LC is frequently underestimated. This is best exemplified by the light chain derived from variable gene segment IGLV3-21, associated with poor prognosis, independently of IGHV mutational status and stereotype (27, 28).

In particular, we identified the single point mutated IGLV3-21^R110^ as a novel molecular biomarker directly linked to BCR signaling and survival of CLL cells. Notably, crystallographic analyses identified the crucial residues of IGLV3-21^R110^ required for homotypic BCR-BCR interaction leading to autonomous signaling in CLL subset #2 (**Figure 1C**) (27, 44). Critical residues include a non-synonymous mutation in the IGLJ segment of the IGLV3-21 derived LC, introducing an indispensable arginin at the position 110 (R110). Of note, IGLV3-21 is the only LC variable gene segment which encodes the required D residues within the CDR2 region, for the homotypic interaction (**Figure 1C**). Among the three major IMGT annotated alleles of IGLV3-21 locus, only allele IGLV3-21*01 encodes the prerequisite K16 and YDSD motif in CDR2. Notably, a recent update in IMGT reference directory (release 202018-4) includes IGLV3-21*04, which also fulfills these requirements. Thus, the allelic variants IGLV3-21*01 or IGLV3-21*04 have intrinsic potential for the generation of autonomously active BCRs. Especially, B cells expressing the autonomously signaling IGLV3-21^R110^ variant are counter-selected in healthy donors (HDs) (27). Although IGLV3-21 is commonly found in association with its heavy chain counterpart IGHV3-21 in stereotyped subset #2 CLL, recent studies confirmed the prognostic value of IGLV3-21^R110^ beyond the IGHV identity and epigenetic classifications of CLL (26, 27, 42, 44). This prompted the idea of a novel subgroup of aggressive CLL expressing the mutated IGLV3-21^R110^, demonstrating the importance of the light chain as an inclusive criteria for CLL prognosis (26, 27). Notably, the recent finding of higher-order correlation between several major and minor CLL subsets are in line with the IGLV3-21^R110^ based broader classification which is best exemplified by CLL subset #2 and #169 (27, 41). Both stereotyped subsets employ the IGLV3-21^R110^ light chain and display structural and immunogenetic similarities with severe clinical courses suggesting a common antigen selection process in their pathogenesis (45). Alternatively, the homotypic BCR-BCR interaction caused by IGLV3-21^R110^ and subsequent autonomous signaling promote the clonal expansion (27). Given the important role of IGLV3-21^R110^, we developed two monoclonal antibodies, one to detect the expression of the functional biomarker IGLV3-21^R110^ and one to distinguish it from the unmutated founder LC. Indeed, the monoclonal anti-wild-type IGLV3-21 antibody can recognize the expression of the susceptible allele IGLV3-21*01 in HDs which is regarded as a risk index for CLL development. Altogether, the discovery of IGLV3-21^R110^ in severe CLL cases, the related mechanism of B cell activation and the development of antibody-based immunophenotyping tools established IGLV3-21^R110^ as functionally linked biomarker for CLL pathogenesis.

## The Stochastic Process of Neoplastic B Cell Transformation and Gain-of-Autonomous Signaling Through Immunoglobulin Mutations

**Figure 2:**
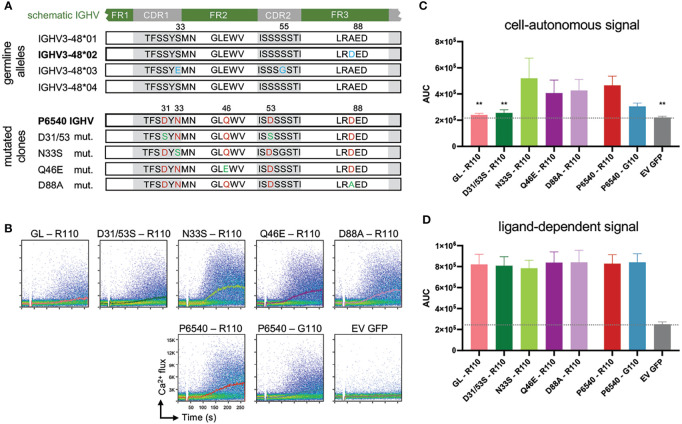
Specific IGHV associated point mutations of autonomous active BCR affect cell-autonomous signal. **(A)** In the upper part sequence alignment of 4 identified germline alleles of IGHV3-48 are shown, differences are marked in blue. Allele 01 and 04 are identical. In the lower part sequence alignment of CLL-patient P6540 derived IGHV is displayed, which is related to IGHV3-48*02 allele (Mutations marked in red). Several single point-mutations were reverted to their germline version, depicted in green. **(B-D)** IGHV and IGLV sequences obtained from the CLL patient P6540 were cloned into retroviral expression vectors for human μHC and λLC and expressed in the TKO system (see **Supplementary Material and Method**). Proper BCR expression on the TKO cells was assessed *via* flow cytometry (data not shown). Germline IGHV3-48*02 (GL) was included for comparison. IGHVs were co-expressed with autonomously active subset #2 IGLV3-21^R110^ LC variant. As control, P6540 IGHV was expressed with IGLV3-21^G110^. Indo-1 staining was performed to analyze intracellular calcium release. In particular, cell-autonomous signaling was assessed *via* calcium mobilization after 4-Hydroxytamoxifen administration, **(B)** shown in representative dot plots with kinetics and **(C)** statistically summarized as area under the curve of the kinetic (AUC). **(D)** As control B-cell receptors were additionally stimulated with anti-LC antibody to mimic ligand-dependent signal. One-Way ANOVA (Bonferroni Correction), significance of mean to mean of P6540 R110 is shown, (**p < 0,01) N = 5.

The process of neoplastic transformation in CLL and other B cell malignancies is controversial. Recent evidence proved the existence of susceptible alleles and mutations which might act as the main drivers of neoplastic transformation (46, 47). Conversely, clonal tracking of leukemic cells indicate that mutations arise at different stages prior and during the course of the disease leading to different cell fates depending on the affected genes (16, 48, 49). Moreover, clonal diversity analyses of IGHV-IGHD-IGHJ sequences revealed the continuous occurrence of mutations regardless of the initial mutation load in the parental (clinically dominant) leukemic clone (50). In short, the rate of intraclonal diversification is similar in UM- and M-CLL. Although this particular model suggests an AID-independent, non-canonical mode of somatic hypermutation, we and others found AID overexpression in M-CLL cases and in IGLV3-21^R110^ cases (27, 51, 52). The off-target effect of such non-canonical SHM results in non-IG genomic mutations (53, 54). When combined with the strength of BCR and other survival signals, the newly induced non-IG genomic mutations persist and clonally propagate. Alternatively, genomic mutations themselves deliver survival advantage in combination with structurally altered FRs of mutated BCRs allowing sustained signaling. In this context, we demonstrate that the survival signal is sustained by the gain-of-autonomous BCR signaling attained through point mutations in IGLV and IGHV genes (**Figure 2**) (27). Thus, we propose a stochastic transformation process, which continuously evokes new mutations that break-out the growth and survival dependencies and overrule the intrinsic DNA damage repair system (**Figure 1C**) (55, 56).

As described before, IGLV3-21^R110^ is originally identified in stereotyped CLL subset #2, which represents a BCR isotype IgM/λ encoded by the IGHV3-21/IGLV3-21 variable gene segments displaying distinctive SHMs (44). Nevertheless, we showed that IGLV3-21^R110^ LCs are compatible with a range of mutational status and different IGHV identities including IGHV3-48 derived IgM-BCRs (26, 27). Notably, stereotyped CLL subset #2 has eight related immunogenetic subsets, defined as satellite subsets, with the most frequent being CLL subset #169 (41). Intriguingly, multiple features of CLL subset #2, including IGHV mutational status, CDR3 length and mutated IGLV3-21^R110^ association, are adopted in the major satellite subset #169, e.g., case P6540 (45). Remarkably, P6540 derived IGHV sequence annotates to mutated IGHV3-48*02 among 4 major IMGT IGHV3-48 alleles (**Figure 2A**). Apart from the acquired LC mutation resulting in IGLV3-21^R110^, the IGHV sequence introduces three critical acidic D residues in each of CDR1, CDR2 and framework region 3 (FR3) regions. Considering P6540 as a model, we examined the combined effect of IGHV and IGLV mutations on autonomous BCR signaling. Using a bifluorescent expression system, the original P6540 derived IgM-BCR and its IGHV mutants were expressed and analyzed for Ca^2+^ influx in triple knockout (TKO) cell system, as described before (57). Interestingly, complete reversion of the IGHV3-48 segment to its germline version ceases the autonomous signaling, despite the use of the IGLV3-21^R110^ light chain (**Figures 2B, C**). Similar result is found in double mutant D35/53S replacing each of CDR1 and CDR2-bound aspartic acid (D) residues with serine (S). Therefore, the acquired point mutations in the CDR1 and CDR2 regions of the IGHV3-48 segment are crucial for the autonomous signaling of the P6540 BCR (**Figures 2B, C**), but irrelevant for ligand-dependent signaling (**Figure 2D**).

Interestingly, both subset #2 and its satellite subset #169, as IGLV3-21^R110^ CLL expressing cases, exhibit high frequency genomic aberrations in regulators of DNA damage response, including splicing factor *SF3B1* (41, 49). Furthermore, we found that in non-subset #169 IGHV3-48 associated cases mutations also accumulate in *TP53*, *ATM*, chromatin modifying *EP300*, and PI3K signaling associated phosphatase *PTEN* (**Figure 1C**) (27). These recurrent mutations often arise in chemotherapy resistant patients (9, 16, 22, 58). Specifically, *TP53* and *ATM* mutations are associated with poor outcome in CLL. Indeed, B lineage specific deletion of *TP53* and *ATM* in Eµ-TCL-1 mice model results in earlier disease onset and more aggressive leukemic features compared to wild-type littermates (20–23). Therefore, recurrent mutations rejuvenate the malignant growth and contribute to the neoplastic transformation but may not resemble the primary transforming factor. On the contrary, B cell specific double transgenic mice overexpressing anti-apoptotic Bcl-2 and dominant negative form Traf2 adapter develop CLL/SLL-like phenotype with restricted IGHV usage mimicking CLL BCR stereotypes (59). This model suggests a conducive role of anti-apoptosis, allowing either selection of BCR clones from an indolent pool or escape of autoreactive clones from the self-elimination process. Thus, either aberrant BCR signaling or the compounding effect of *ad hoc* signaling crosstalk are the foremost transforming factor. On top, the appearance of further recurrent mutations in genes such as *SF3B1* and *TP53* accelerate and exacerbate the disease when combined with permissive BCR signaling or other conducive features - resembling a stochastic process.

## Discussion

With the advent of GWAS and other NGS approaches, we witnessed the expansion of the previously used criteria for CLL prognosis and the introduction of new CLL-IPI classification (24, 25). Nevertheless, the CLL-IPI classification requires extensive genetic and molecular characterization (*TP53* and IGHV) limiting a quick assessment. In contrast, novel diagnostic tools exploiting the existence of functionally relevant biomarkers delivers more quicker and easier assessment. To this aim, we developed monoclonal antibodies for specific detection of the point-mutated light chain IGLV3-21^R110^, which with appropriate and extensive validation could represent an easy and quick diagnostic tool for severe CLL cases. The presence of IGLV3-21^R110^ positive B cells or the expression of unmutated IGLV3-21 from susceptible allele IGLV3-21*01 in healthy donors (HD) is regarded as a risk index for CLL development (26–28). For early diagnosis and preemptive therapy, IGLV3-21^R110^ is a gem and we foresee future development of antibody-based direct cell-depletion or drug delivery interface. Additionally, quick immune-detection of unmutated IGLV3-21 LC expression combined with genetic analyses of IGLV3-21 allele in HD will predict the susceptibility to severe CLL.

Innovative genomic studies pointing out the existence of risk-associated loci and novel murine models of genomic alteration such as *Sf3b* mutation and *ATM* deletion, prompted the idea of a stochastic process of malignant transformation where the expression of a susceptible allele or mutations are regarded as the driver event for future development of neoplastic diseases. The existence of risk-associated loci also highlights the dynamics of familiar CLL, confirming that relatives of CLL patients have an increased risk of developing the disease (60, 61). In particular, CLL cases within the same family presented uniform BCR IG and similar genomic profiles, highlighting the correlation between germline predisposition and immunogenetic markers and pointing towards a shared mechanism of CLL development (62). Of note, recurrent unfavorable mutations are often associated with survival and anti-apoptotic pathways or with BCR signaling pathway. It is important to understand the role of these mutations as prognostic indicators, not only to improve diagnosis, but also to develop suitable preclinical models, which comprehend the heterogeneity of CLL.

So far, the majority of CLL mouse models mimic genetic aberrations or deregulated gene expression in CLL without taking into account the clonality of the BCR (63). Nevertheless, in a stochastic process of malignant transformation, cells accumulate numerous mutations which do not proliferate until the occurrence of additional events that overcome the checkpoints and drive the leukemogenesis. Thus, the generation of an inducible monoclonal CLL-mouse model to study CLL development in a stepwise manner would be of particular relevance to clarify the role of aberrant BCR signaling in the development of CLL. We propose that the use of a CLL-derived autonomous BCR, e.g. the aggressive subset #2 will help to elucidate the course of CLL. In fact, it is interesting to follow the growth of subset #2 BCR expressing B cells, preferably in inducible manner, *in-vivo* and to screen for acquired mutations. Moreover, subsequent changes in anti-apoptotic and pro-survival properties of these autonomously active B cells are of immense relevance for both indolent and aggressive outcome. Thus, an inclusion of functionally relevant prognostic parameters, even for indolent cases, can only be beneficial. Of course, further and larger independent studies must be carried out in order to validate these parameters.

Characterization of the molecular prognostic parameters and genomic landscape predict primary response but not relapse to treatment. Often, the underrated interactions between molecular cytogenetics features and the microenvironment specific survival signals mediated by direct contacts, adhesion molecules, chemokines and ligand-receptor interactions are of great prognostic value (15, 64–66). The kinases of BCR signaling pathway such as PI3K, BTK and SYK, are also involved in receptor mediated crosstalk (67). For example, *ibrutinib*, the BTK inhibitor, interferes with the homeostasis of the leukemic cells in the survival niches, thus demonstrating efficacy in high-risk CLL (22, 64, 68–70). Moreover, tissue infiltrating and colonizing ability of leukemic cells alters growth rate, metabolic dependency and proliferation of CLL cells (71). Recent studies have highlighted the importance of analyzing CLL cells in a physiologically relevant environment recreating the interactions between leukemic cells and their milieu (64). Such 3D-cell culture and bioreactor models analyze the role of the microenvironment comprehensively. This is of great resonance to characterize tumor cells in their milieu providing novel *in vitro* strategies to test new therapeutic agents and assess their effects under more *in vivo*-like conditions.

## Data Availability Statement

The raw data supporting the conclusions of this article will be made available by the authors, without undue reservation.

## Author Contributions

PCM designed the study, led the experiments and interpreted the results together with HJ. AN and ATL performed the experiments, wrote the MS with PCM, and all authors reviewed, commented on, and approved the manuscript.

## Funding

This work was supported by SFB 1074 (A10), SFB 1279 (B03) and the ERC 694992 to HJ; AN is PhD student through SFB1279; ATL is postdoctoral fellow through ERC 694992; PCM is research fellow at Institute of Immunology, Ulm University.

## Conflict of Interest

The authors declare that the research was conducted in the absence of any commercial or financial relationships that could be construed as a potential conflict of interest.

## Publisher’s Note

All claims expressed in this article are solely those of the authors and do not necessarily represent those of their affiliated organizations, or those of the publisher, the editors and the reviewers. Any product that may be evaluated in this article, or claim that may be made by its manufacturer, is not guaranteed or endorsed by the publisher.
