# Supplementary material for: The Determinants of B Cell Receptor Signaling as Prototype Molecular Biomarkers of Leukemia
Source: Front Oncol. 2021 Dec 21;11:771669. doi: 10.3389/fonc.2021.771669 (PMC8724047; doi:10.3389/fonc.2021.771669)
Supplement: Supplementary file 1 [file DataSheet_1.pdf]

Supplementary Materials and Methods

Cell culture

Phoenix and triple knockout (TKO) cells were cultured in Iscove medium (Sigma) containing 10% FCS (PAN-Biotech), 10 mM L-glutamine (Life Technologies), 50 mM ß-Mercaptoethanol (Life Technologies), and 100 U/ml each of penicillin and streptomycin (Life Technologies). For TKO cells the medium was supplemented with 1 ng/ml recombinant murine IL-7 (Immunotools).

Plasmids and retroviral transduction

IGHV and IGLV sequences obtained from the CLL patient P6540 were cloned into retroviral expression vectors for human µHC and λLC flanked by an internal ribosomal entry sequence followed by split-green fluorescent protein (GFP) reporters as described previously (Iype, 2019; Maity, 2020). Germline sequence IGHV3-48*02 was retrieved from IMGT. Retroviral transduction was performed as described previously (Köhler, 2008; Meixlsperger, 2007). Briefly, phoenix cells were transfected with µHC- and λLC-plasmids using GeneJuice (Novagen) as recommended by the manufacturers protocol and culture medium containing the secreted retrovirus was collected after 2 days of transfection. TKO cells expressing ERT2-SLP65 were transduced with retrovirus supernatant by spin-infection method and GFP-expressing BCR positive cells were analyzed three to five days after transduction.

Measurement of Ca2+ flux

Ca2+ mobilization analyses were performed as described (Iype, 2019; Maity, 2020). Briefly, a total of 1 million transduced TKO cells expressing ERT2-SLP65 were loaded with Indo-1 AM (Invitrogen) using Pluronic F27 (Invitrogen). ERT2-SLP65 function was induced by the addition of 2 µM 4-hydroxy tamoxifen (4-OHT) (Sigma-Aldrich).

Statistical analysis

Data were analysed with FlowJo 10. Area under the curve (AUC) and kinetics were calculated by the software. Statistical analysis was performed with Prism 8. One-Way ANOVA with Bonferroni Correction was performed. Significance of mean to mean of P6540 R110 is shown (**p<0,01).

1. P.C. Maity, M. Bilal, M.T. Koning, M. Young, C.A.M. van Bergen, V. Renna, A. Nicolò, M. Datta, E. Gentner-Göbel, R.S. Barendse, S.F. Somers, R.A.L. de Groen, J.S.P. Vermaat, D. Steinbrecher, C. Schneider, E. Tausch, T. Bittolo, R. Bomben, A.N. Mazzarello, G. Del Poeta, W.G.M. Kroes, J.T. van Wezel, K. Imkeller, C.E. Busse, M. Degano, T. Bakchoul, A.R. Schulz, H. Mei, P. Ghia, K. Kotta, K. Stamatopoulos, H. Wardemann, A. Zucchetto, N. Chiorazzi, V. Gattei, S. Stilgenbauer, H. Veelken, and H. Jumaa, IGLV3-21*01 is an inherited risk factor for CLL through the acquisition of a single-point mutation enabling autonomous BCR signaling. Proc Natl Acad Sci U S A 117 (2020) 4320-4327.
2. J. Iype, M. Datta, A. Khadour, R. Übelhart, A. Nicolò, T. Rollenske, M. Dühren-von Minden, H. Wardemann, P.C. Maity, and H. Jumaa, Differences in Self-Recognition between Secreted Antibody and Membrane-Bound B Cell Antigen Receptor. J Immunol 202 (2019) 1417-1427.
3. F Köhler, E Hug, C Eschbach, S Meixlsperger, E Hobeika, J Kofer, H Wardemann, and H Jumaa, Autoreactive B Cell Receptors Mimic Autonomous Pre-B Cell Receptor Signaling and Induce Proliferation of Early B Cells. Immunity 29 (2008) 912–921.
4. S Meixlsperger, F Köhler, T Wossning, M Reppel, M Müschen, and H Jumaa, Conventional Light Chains Inhibit the Autonomous Signaling Capacity of the B Cell Receptor. Immunity 26 (2007) 323–333.
